# Description of two new species of the genus *Cacopsylla* Ossiannilsson, 1970 (Hemiptera, Psylloidea) from northern Fennoscandia recognized by morphology, cytogenetic characters and *COI* barcode sequence

**DOI:** 10.3897/CompCytogen.v13i4.47395

**Published:** 2019-11-19

**Authors:** Christina Nokkala, Valentina G. Kuznetsova, Veikko Rinne, Seppo Nokkala

**Affiliations:** 1 Laboratory of Genetics, Department of Biology, University of Turku, FI-20014 Turku, Finland; 2 Department of Karyosystematics, Zoological Institute, Russian Academy of Sciences, Universitetskaya nab. 1, 199034 Saint Petersburg, Russia; 3 Zoological Museum, University of Turku, FI-20014 Turku, Finland

**Keywords:** *
Cacopsylla
*, *COI*, karyotype, morphology, new species, Northern Europe, bisexual reproduction, parthenogenesis

## Abstract

Based on chromosomal, molecular and morphological analyses, two new *Cacopsylla* Ossiannilsson, 1970 species are described, *C. lapponica* S. Nokkala & Ch. Nokkala, **sp. nov.** and *C. borealis* S. Nokkala et Ch. Nokkala, **sp. nov.** (Hemiptera, Psylloidea). *C. lapponica* is a rare bisexual alpine species living on *Vaccinium uliginosum* Linnaeus, 1753 above tree line on northern hills, where it forms sympatric populations with *C. myrtilli* W. Wagner, 1947. So far, the species has been found in northern Finland, Utsjoki and Kilpisjärvi, and in northern Sweden, Abisko. The chromosome number in males is 2n = 12+X(0), characteristic of psyllids. The species is easily distinguished from *C. myrtilli* by its conspicuously smaller size mainly due to difference in wing size. Additional morphological differences are found in the length of antennae, female genital plates and male parameres. *C. borealis*, in turn, is a relatively common apomictic parthenogenetic species with 5n = 60 + XXXXX living on the same host plant, *Ledum palustre* Linnaeus, 1753, as *C. ledi* (Flor, 1861) and occasionally forming sympatric populations with it. No males have been recorded in *C. borealis*. Its distribution range reaches at least from northern Fennoscandia to Lake Baikal in the East. *C. borealis* can be distinguished from *C. ledi* by differences in the length and width of antennae, dark brown markings on the wing and female terminal structures. For molecular analysis, a 638 bp fragment of the mitochondrial *COI* gene was sequenced. *C. lapponica* differs from the cohabitating *C. myrtilli* by 20 fixed nucleotide substitutions (uncor rected p-distance 3.13 %), while *C. borealis* differs from *C. ledi* by 21 fixed nucleotide substitutions (uncorrected p-distance 3.29 %). Molecular phylogeny construction (ML and BI) reveals two highly divergent clades, one comprising two bisexual species, *C. lapponica* and *C. fraudatrix* Labina & Kuznetsova, 2012, and the other clade comprising the parthenogenetic species *C. borealis*, *C. myrtilli*, and *C. ledi*. Within this clade, *C. borealis* is more closely associated with *C. myrtilli* than with *C. ledi*.

## Introduction

It is well established that in Northern Europe two Holarctic psyllid species, *Cacopsylla myrtilli* (W. Wagner, 1947) and *C. ledi* (Flor, 1861), inhabit the host plants *Vaccinium myrtillus* Linnaeus, 1753 or *V. uliginosum* Linnaeus, 1753 and *Ledum palustre* Linnaeus, 1753, respectively (Ossiannilsson 1992). Both species are widely distributed through the temperate and alpine zones in Fennoscandia, Central Europe and Russia. *C. ledi* is quite evenly distributed throughout the distribution range, while the distribution of *C. myrtilli* is concentrated towards the north and/or high altitudes. Females of both species are triploid and reproduce through apomictic parthenogenesis (Ossiannilsson 1992; Nokkala et al. 2008, 2017). Quite commonly, infrequent males known as rare males exist in their populations. Males are mainly nonfunctional in populations of *C myrtilli* (Nokkala et al. 2013) and functional in *C. ledi* populations (Nokkala et al. 2017). In populations with rare males, infrequent diploid females also exist among the triploids (Nokkala et al. 2015).

So far, bisexual diploid ancestral species from which parthenogenetic *C. myrtilli* and *C. ledi* are evolved are still unknown. Klimaszewski (1971) reported a bisexual *C. myrtilli* population from the Bieszczady Mountains in Poland. However, based on *COI* barcoding DNA sequence and testis structure, Kuznetsova et al. (2012) showed the *Cacopsylla* Ossiannilsson, 1970 species living on *V. myrtillus* in Bieszczady to represent a novel bisexual species, *C. fraudatrix* Labina & Kuznetsova, 2012.

The first observation that new species could also be found in Northern Europe was made in Abisko, northern Sweden. While sampling a *C myrtilli* population near Lapporten at 570 m altitude above the tree line, three males were caught. Unlike in *C. myrtilli* which showed absence of chiasmata in male meiosis (Nokkala et al. 2013), chiasmata were present in meiosis in these males. *COI* sequence of the males was quite different from that found in *C. myrtilli*. Similarly, we found *Cacopsylla* individuals on *L. palustre* displaying *COI* sequence quite different from that of *C. ledi* but utilizing the same host plant.

As a result, we describe two new *Cacopsylla* Ossiannilsson, 1970 species which vary in their external morphology, karyotype and *COI* sequence from *C. myrtilli* and *C. ledi*, respectively.

## Material and methods

### Sampling

*Cacopsylla* specimens were collected in various locations in Sweden, Finland and Russia (Table 1). A part of the individuals was brought alive to the laboratory for morphological analysis, while most specimens were stored in alcohol or fixative (see below).

### Cytological methods

In most cases whole individuals were fixed immediately after collecting in 3:1 (ethanol: acetic acid) fixative. A part of the insects was dissected, and the abdomen was immersed in fixative for cytological analysis while the head and thorax were stored in ethanol for molecular analysis of the same individual. Cytology was performed as described by Nokkala et al. (2008) with 30 min Schiff staining and 40 min Giemsa staining.

Chromosome preparations were photographed with Nikon DS-Fi3 camera mounted on Nikon Ci-L microscope using NIS Elements software. Final processing of photomicrographs was made with Corel-PhotoPaint 2018 software.

### DNA isolation, amplification and sequencing

Genomic DNA was isolated from whole insects or from the head and thorax portion of individuals stored in alcohol using the DNeasy Blood and Tissue Kit (Qiagen) as described previously (Nokkala et al. 2015, 2017). A fragment of the mitochondrial *COI* gene was amplified using the primer pair HybCacoCO / HybHCOMod. PCR reactions were carried out in 20 µl volume containing 1 x PCR buffer, 2.0 mM MgCl_2,_ 200 µM dNTP each, 0.5 µM of forward and reverse primers, 0.5 U DreamTaq DNA Polymerase (ThermoFisher Scientific) and 1 µl (ca. 50 ng) of template DNA. Initial denaturation in PCR reaction was 5 min at 95 °C followed by 40 cycles in 95 °C (30 sec), 50 °C (30 sec) and 72 °C (90 sec) with a final extension at 72 °C for 10 min. PCR products were purified with QIAquick PCR Purfication Kit (Qiagen) and sent to Macrogen Europe (Amsterdam, the Netherlands) for sequencing. BioEdit 7.2.0 (Hall 1999) software was used to trim the sequences to span a 638 bp fragment of the gene and to align the sequences with related *Cacopsylla* species, *C. fraudatrix* (GenBank accession number JX987970 = h8) (Kuznetsova et al. 2012), *C. myrtilli* (KF494326–KF494332 = h1–h7) (Nokkala et al. 2015), *C. ledi* (MF978762–MF978766 = h9–h11, h13–h14) (Nokkala et al. 2017) and JX987973 = h12 (Kuznetsova et al. 2012). In addition, we sequenced the same gene region from a related bisexual species *C. corcontum* (Šulc, 1909), accession number MK184915 (= h18), used as an outgroup in phylogenetic analysis. Sequences obtained from new species have been deposited in GenBank under the accession numbers MK184912 (= h15) and MK184913 (= h16) for *C. borealis*, and MK184914 (= h17) for *C. lapponica*.

### Phylogenetic analysis

Tests of the homogeneity of substitution patterns and estimation of the net composition bias disparity between sequences were carried out with MEGAX software (Kumar et al. 2018). We used two approaches for phylogenetic inference, a maximum likelihood method (ML) and a Bayesian inference method (BI). To find the best substitution model for the current data set, MEGAX software was applied and Tamura 3-parameter + G was selected for the ML analysis and GTR+G+I for the Bayesian analysis. ML phylogenetic reconstruction was carried out using MEGAX software. Branch support was assessed with 1000 bootstrap pseudo-replicates. MrBayes 3.2.6 (Ronquist et al. 2012) was used for Bayesian inference. Two runs of the program for 1 000 000 generation, sampled every 1000 generations, were run with four chains (one cold and three heated, with heating value t = 0.2). The first 250 chains were discarded as burn-in prior to computing the consensus phylogeny and posterior probabilities (burn-infrac = 0.25). Model parameters were treated as unknown and were estimated during analysis.

### Morphology

The number of testicular follicles was determined as described by Kuznetsova et al. (2012).The overall length, genital marker qualities and distribution of surface spinules in the c+sc cell of the forewing were determined in females and males (if available). Photomicrographs were taken with a Canon EOS 7D camera attached to an Olympus SZX16 stereomicroscope at the Zoological Museum, University of Turku, Finland. The camera was driven by a QuickPHOTO MICRO 3.1 software. Images were stacked using Zerene Stacker and CombineZP software.

## Results

### Cytology

**Figure 1.**
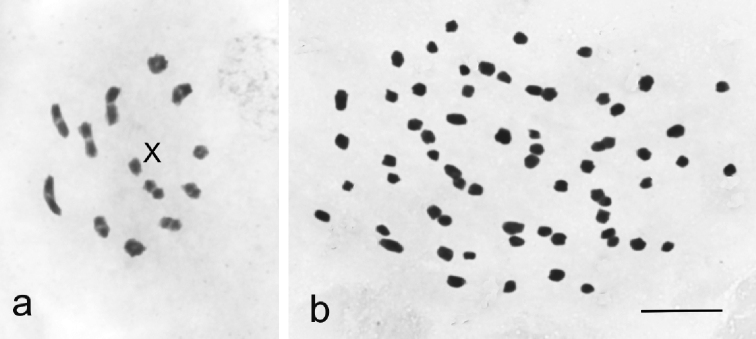
Chromosomes in *C. lapponica* sp. nov. and *C. borealis* sp. nov. **a** Metaphase I in male meiosis in *C. lapponica* sp. nov. showing 12 autosomal bivalents and a univalent X chromosome (2n = 24+X(0)) **b** Late prophase from a mature egg in *C. borealis* sp. nov. with 65 univalent chromosomes (5n = 60+5X). Scale bar: 10 µm.

In *C. lapponica* sp. nov. populations, males and females were present in equal numbers (Table 1), hence this species reproduces bisexually. Testes in males consisted of two follicles and meiosis was chiasmate showing 12 bivalents and a univalent X chromosome at metaphase I (Fig. 1a). In the populations of *C. borealis* sp. nov. no males were found (Table 1) indicating that females reproduce parthenogenetically. In mature or nearly mature eggs, 65 univalent chromosomes were present at prometaphase, hence, females were apomictic and pentaploid (Fig. 1b).

**Table 1.** *Cacopsylla* material morphologically analyzed (number of individuals sequenced).

**Species**	**Country**	**Locality**	**N**		**Cooordinates**	**Altitude (m)**	**Food plant**	**Date**	**Collector**
			**females**	**males**	**Lat. / Long.**				
*Cacopsylla lapponica* sp. nov.	Sweden	Abisko	19 (2)	3 (3)	68°19'14", 18°51'05"	570	*V. uliginosum*	10.8.2012	S. & Ch. Nokkala
	Finland	Utsjoki, Ailigas	8 (1)	7 (1)	69°53'51", 27°03'32"	320	*V. uliginosum*	6.8.2016	S. & Ch. Nokkala
		Kilpisjärvi	5	7	69°03'50", 20°44'20"	620	*V. uliginosum*	27.7.2014	S. & Ch. Nokkala
			3 (2)	3 (1)				5.8.2016	S. & Ch. Nokkala
*Cacopsylla borealis* sp. nov.	Sweden	Muodoslompolo	97 (4)	0	68°11'13", 23°00'20"		*L. palustre*	9.8.2018	S. & Ch. Nokkala
	Finland	Utsjoki, Hietala	87 (3)	0	69°51'06", 27°00'34"		*L. palustre*	15.8.2017	S. & Ch. Nokkala
		Utsjoki, Ailigas	148 (12)	0	69°53'51", 27°03'32"	320	*L. palustre*	6.8.2016	S. & Ch. Nokkala
			54 (3)	0				15.8.2017	S. & Ch. Nokkala
		Inari, Pitkävuono	117 (5)	0	68°59'56", 26°58'48"		*L. palustre*	16.8.2017	S. & Ch. Nokkala
		Sodankylä, Puisuvanto	165	0	67°46'52", 26°46'09"		*L. palustre*	3.8.2018	S. & Ch. Nokkala
		Salla, Tuntsa	292	0	67°18'11", 29°16'18"		*L. palustre*	1.8.2019	S. & Ch. Nokkala
		Salla, Niemelä	413	0	66°35'48", 28°59'35"		*L. palustre*	2.8.2019	S. & Ch. Nokkala
		Kuusamo, Kantojoki	285	0	66°14'23", 29°09'15"		*L. palustre*	2.8.2019	S. & Ch. Nokkala
		Kuusamo, Sakkojoki	86	0	65°32'02", 29°32'03"		*L. palustre*	3.8.2019	S. & Ch. Nokkala
		Toholampi	19	0	63°45'04", 24°11'58"		*L. palustre*	10.8.2018	S. & Ch. Nokkala
	Russia	Vorkuta	7 (7)	0	67°30'00", 64°02'00"		*L. palustre*	6.8.2013	N. Khabasova
		Baikal	71 (5)	0	51°54'25", 105°04'14"		*L. palustre*	21.7.2007	E. Labina

### Phylogenetic analysis

We obtained partial mitochondrial *COI* gene sequences from five females and five males from three populations of *C. lapponica* sp. nov. and from 41 females from seven populations of *C. borealis* sp. nov. (Table 1). From the outgroup species *C. corcontum*, two sequences were obtained. All *C. lapponica* sp. nov. individuals sequenced shared the same haplotype. In *C. borealis* sp. nov. the same haplotype was found in all specimens from six populations, while the sequences obtained from Vorkuta specimens differed from this haplotype by two substitutions. Additional sequences retrieved from the GenBank, all sequences were trimmed to cover a 638 bp fragment of the gene. The alignment thus included 18 sequences. No indels or stop codons were present. In the alignment, a total of 120 variable sites, 39 of them being parsimony informative, were included.

**Figure 2.**
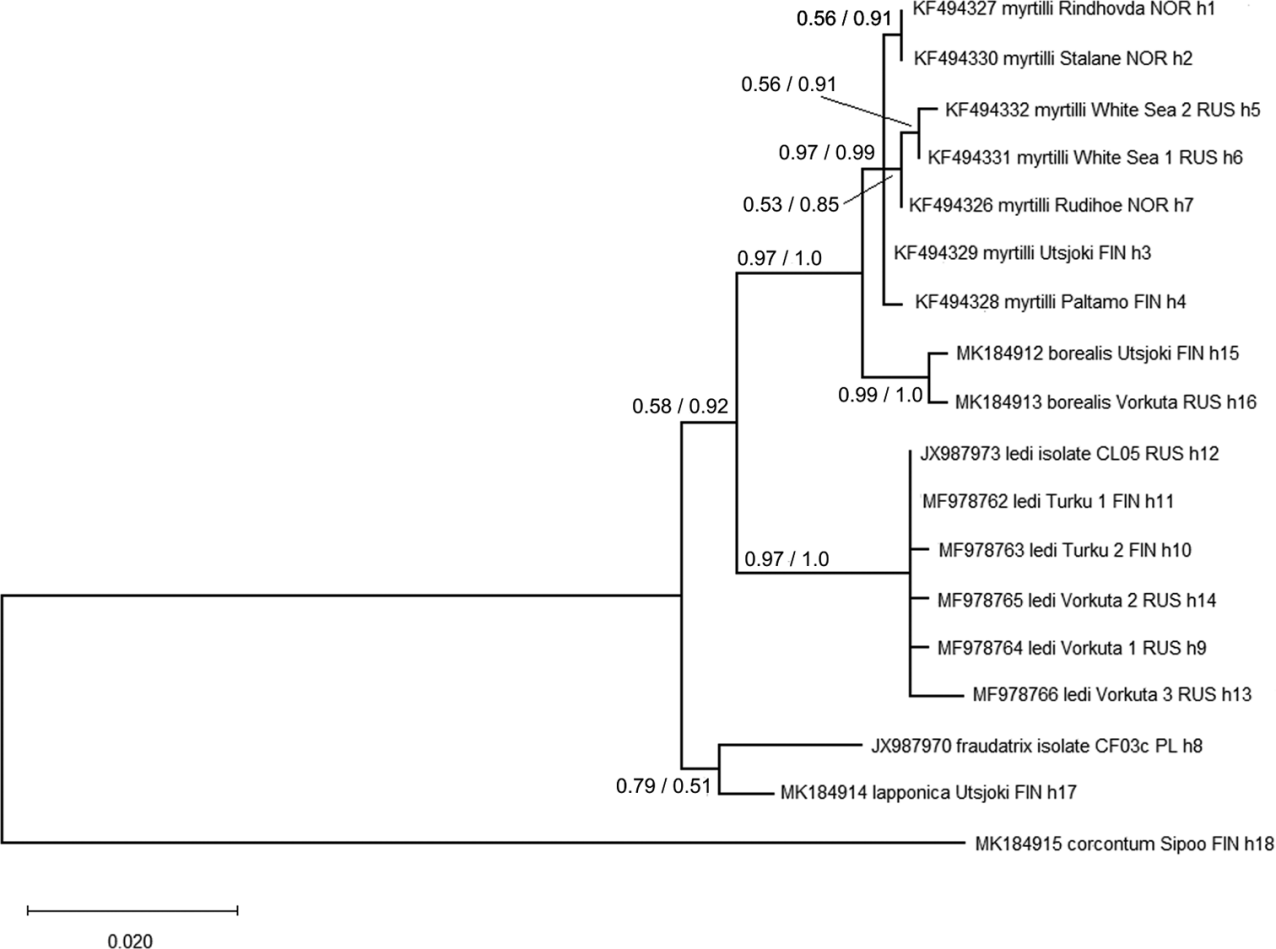
Phylogenetic relationships of *C. lapponica* sp. nov. and *C. borealis* sp. nov. with closely related *Cacopsylla* species. The maximum likelihood bootstrap values /Bayesian posterior probabilities are shown at nodes. Geographical abbreviations: FIN – Finland, NOR – Norway, PL – Poland, RUS – Russia.

ML and Bayesian reconstructions produced trees with identical topology and similar branch support (Fig. 2). The tree included two highly divergent clades, one consisting of parthenogenetic *C. borealis* sp. nov., *C. myrtilli* and *C. ledi*. The other clade included bisexual *C. lapponica* sp. nov. and *C. fraudatrix*. Although *C. borealis* lives on the same host plant as *C. ledi* and forms mixed populations with it, *C. borealis* is more closely associated with *C. myrtilli*. There are only 9 nucleotide substitutions between *C. borealis* sp. nov. and *C. myrtilli*, uncorrected p-distance being 1.41 %, while 13 substitutions have occurred between *C. lapponica* sp. nov. and *C. fraudatrix*, reflected in an uncorrected p-distance of 2.04% (Table 2). The remaining pairwise substitutions fall between 20–23 and uncorrected p-distances between 3.13–3.61%.

**Table 2.** Fixed nucleotide substitutions and uncorrected p-distances between *Cacopsylla* species in the 638 bp mitochondrial *COI* gene fragment. The number of transversions in parentheses ().

	***C. borealis***		***C. myrtilli***		***C. ledi***		***C. lapponica***	
*C. myrtilli*	9 (1)	1.41%						
*C. ledi*	21 (3)	3.29%	20 (4)	3.13%				
*C. lapponica*	22 (3)	3.44%	20 (4)	3.13%	20 (4)	3.13%		
*C. fraudatrix*	23 (3)	3.60%	23 (5)	3.61%	23 (4)	3.61%	13 (3)	2.04%

Together with *C. ledi*, the species *C. borealis* sp. nov. and *C. myrtilli* formed a sister group to the [*C. lapponica* sp. nov. + *C. fraudatrix*] clade.

#### Cacopsylla lapponica

Taxon classificationAnimaliaHemipteraPsyllidae

S. Nokkala & Ch. Nokkala sp. nov.

852BE45B-EDBF-503A-BA01-EF4CAC31553F

http://zoobank.org/65E15B28-AE24-49A8-9026-88A4C1EBE8EC

##### Type material.

***Holotype***: Female; Finland, Utsjoki Ailigas; 69°53'51’’N, 27°03'32’’E; 320 m; 05 Aug 2016; Seppo & Christina Nokkala leg.; above tree line, host *Vaccinium uliginosum*; http://mus.utu.fi/ZMUT.TYPE794. ***Paratypes***: 9 females,1male; Finland, Utsjoki Ailigas; 69°53'51’’N, 27°03'32’’E; 320 m; 05 Aug 2016; Seppo & Christina Nokkala leg.; above tree line, host *Vaccinium uliginosum*; http://mus.utu.fi/ZMUT.TYPE795 – http://mus.utu.fi/ZMUT.TYPE797. The holotype and paratypes are deposited at the Zoological Museum, University of Turku, Finland.

##### Description.

**Figure 3.**
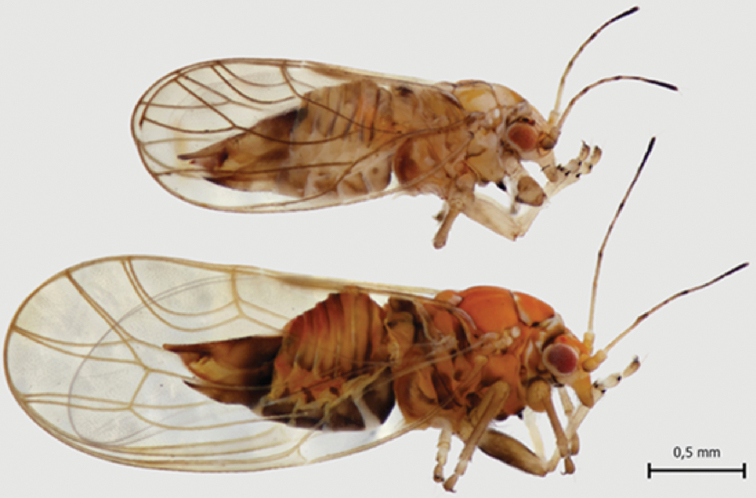
Adult females of *C. lapponica* sp. nov. (above) and *C. myrtilli* (below) showing conspicuously different sized wings.

Adult coloration resembles that of cohabitating *C. myrtilli*, but is much paler with dark markings. Wings are very pale yellow and transparent with dark veins (Fig. 3). Adults are clearly smaller in size (Fig. 3), the overall length of males being 1.9–2.1 mm (N = 8) and females 2.3–2.5 mm (N = 10) compared to 2.75–3.25 mm of *C. myrtilli* females (Ossiannilsson 1992).

##### Diagnosis.

**Figure 4.**
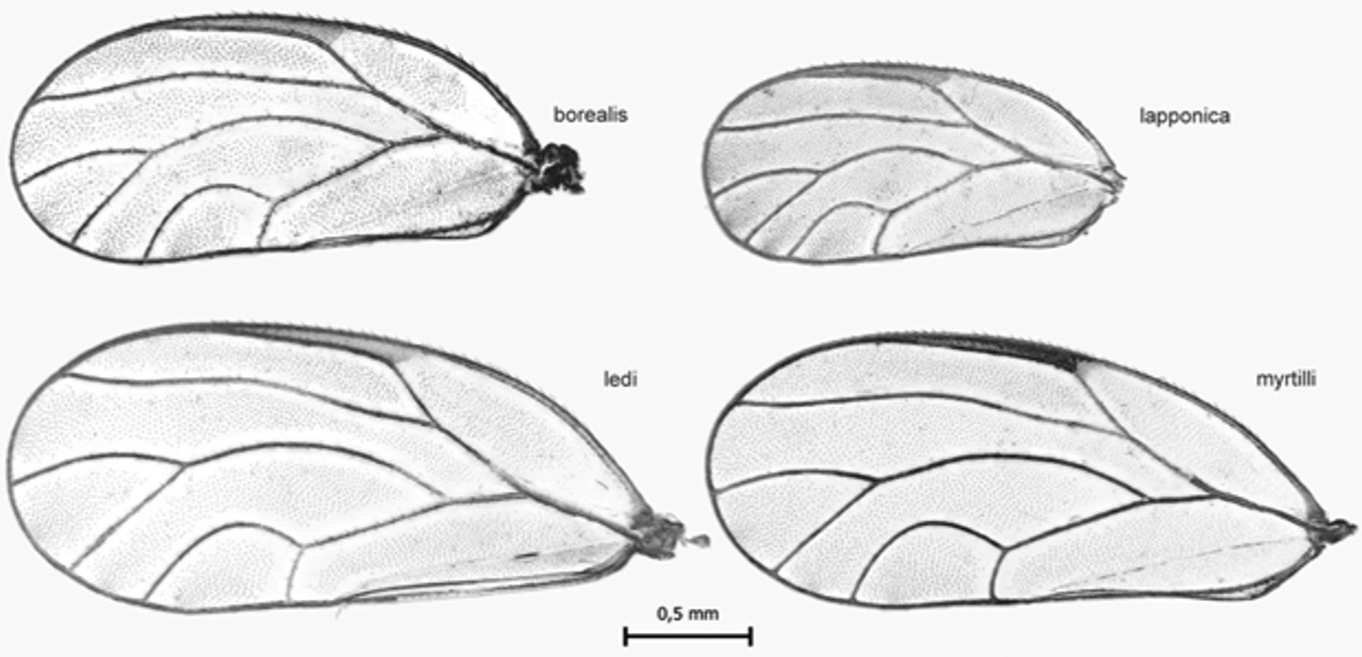
Comparison of forewings in *C. borealis* sp. nov., *C. lapponica* sp. nov., *C. ledi* and *C. myrtilli*.

The most conspicuous difference in external morphology between *C. myrtilli* and *C. lapponica* is the length of wings. In *C. lapponica*, the wing is much shorter than in *C. myrtilli* (Fig. 4). In *C. lapponica*, the wings are just slightly longer than the abdomen, while in *C. myrtilli* the wings are almost twice as long as the abdomen.

**Figure 5.**
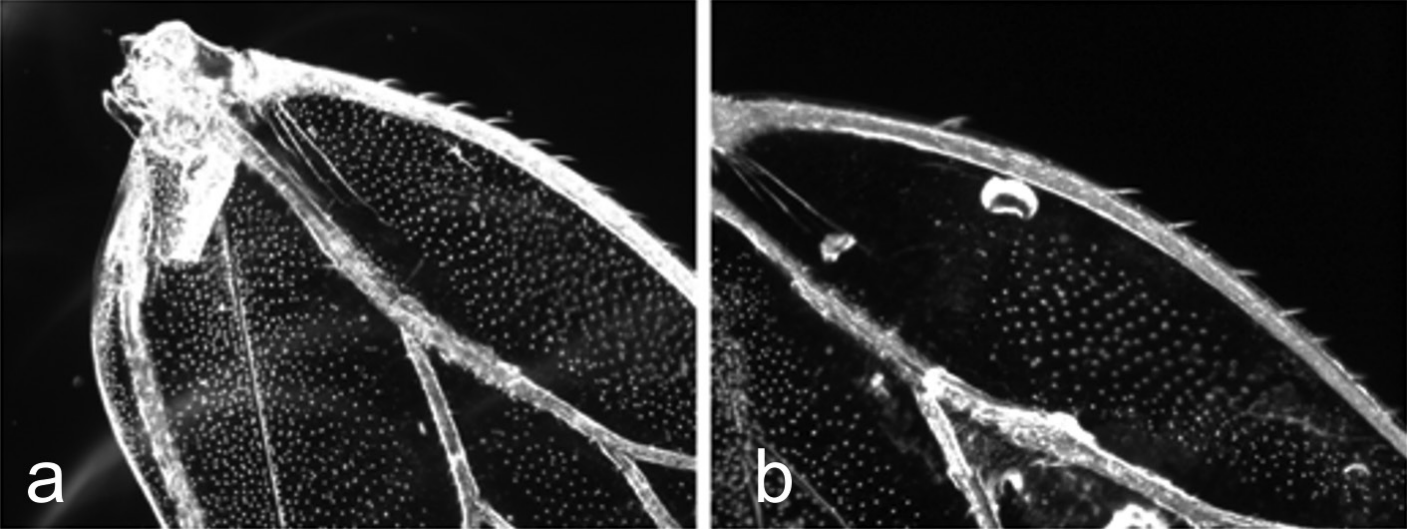
The distribution of surface spinules in the s+cs cell in the forewing **a***C. lapponica* sp. nov. The distribution of spinules is similar to that of *C. myrtilli***b***C. borealis* sp. nov. The distribution of spinules is similar to that of *C. ledi*.

According to the species identification key, the distribution of surface spinules in the s+cs cell in the forewing has been used to separate the closely related species of *C. myrtilli* and *C. ledi* (Ossialnnilsson, 1992). In *C. myrtilli* surface spinules cover the s+cs cell entirely, while in *C. ledi* the spinules are absent in the apical third of the cell. In *C. lapponica* (Fig. 5a), the distribution of spinules is similar to that found in *C. myrtilli*.

**Figure 6.**
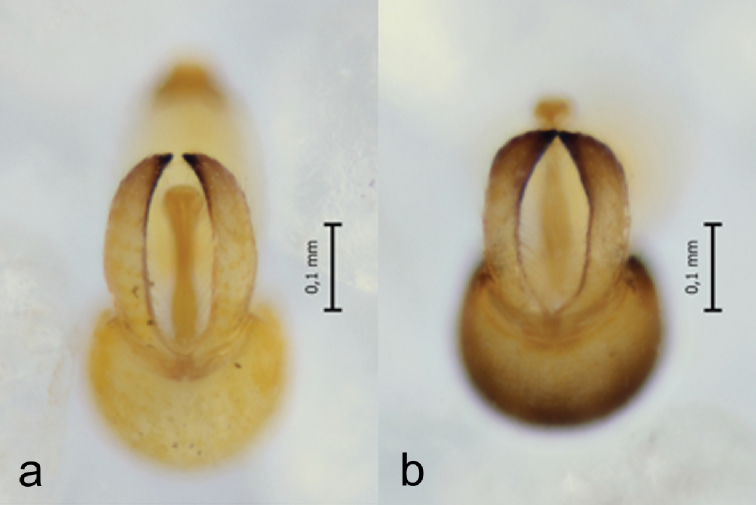
Male parameres from behind **a***C. lapponica* sp. nov. **b***C. myrtilli*.

Males can also be differentiated by their paramere structure (Fig. 6). In males of *C. lapponica*, the thickest region is in the middle of paramere viewed from behind (Fig. 6a), and a similar region is seen in the apical part of paramere in *C. myrtilli* (Fig. 6b).

**Figure 7.**
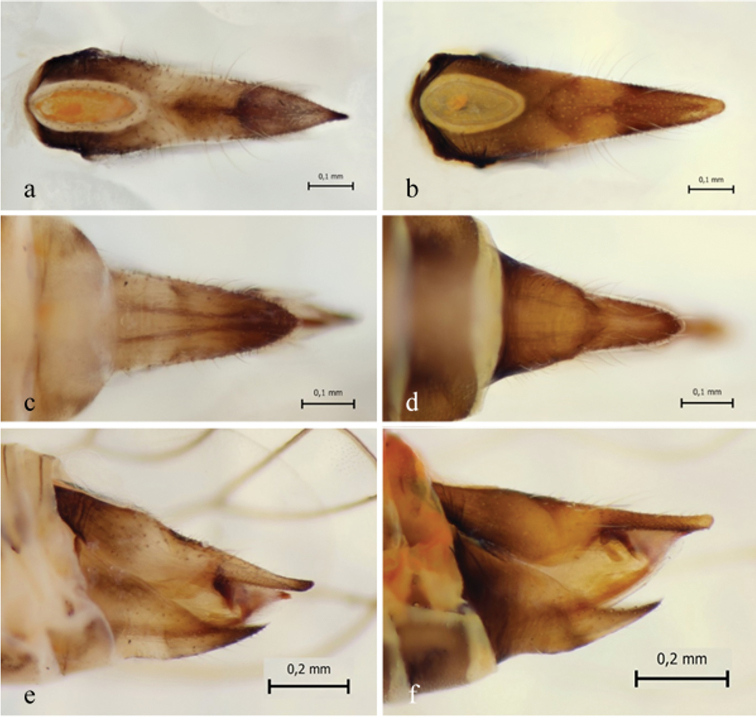
Morphology of female genital structures in *C. lapponica* sp. nov. (**a, c, e**) and *C. myrtilli* (**b, d, f**) **a, b** dorsal plate, proctiger from above **c, d** subgenital plate from below **e, f** genital plates from the left.

Female *C. lapponica* are easily distinguished from *C. myrtilli* females by differences in their terminalia structures (Fig. 7). In *C. lapponica*, the circumanal pore ring complex is symmetric, oval-shaped, and proctiger is sharply pointed (Fig. 7a), whereas in *C. myrtilli*, the same structure is clearly asymmetric and the apical part of proctiger is more rounded (Fig. 7b). As shown below, the subgenital plate evenly decreases in width towards the apex in *C. lapponica* (Fig. 7c), while the width strongly decreased halfway of the plate in *C. myrtilli* (Fig. 7d). In the side view the subgenital plate differs clearly between the species. In *C. lapponica*, the upper edge runs quite straight and is curved only near the apex (Fig. 7e), while in *C. myrtilli*, there is a strong curve already near the middle of the plate (Fig. 7f).

##### Distribution.

Specimens of *C. lapponica* were found in three locations at high altitude above the tree line in northern Sweden and Finland (Table 1). In all these locations, *C. lapponica* coexists with *C. myrtilli* on low growing *V. uliginosum* plants in low numbers. As an example, in a sample collected on 6.8.2016 in Utsojki, Ailigas at 320 m altitude, there were 252 specimens of *C. myrtilli* and among them 15 specimens (8 females and 7 males) of *C. lapponica*, the proportion of *C. lapponica* being 5.6% of the total. It is obvious, that *C. lapponica* is a rare alpine species restricted to a high-altitude open habitat.

##### Etymology.

The name “lapponica” in Latin means “from Lapponia” or “Lapponian” reflecting the restricted distribution of the species to northern Fennoscandia in locations above the tree line.

#### Cacopsylla borealis

Taxon classificationAnimaliaHemipteraPsyllidae

S. Nokkala & Ch. Nokkala sp. nov.

BC4E57BB-1DC3-50DC-8D2F-920D7F0AE019

http://zoobank.org/EB6FC0FC-6BB5-45F4-A4BC-3E88EEAD167A

##### Type material.

***Holotype***: Female; Finland, Salla, Tuntsa; 67°18'11"N, 29°16'18"E; 01. Aug. 2019; Seppo & Christina Nokkala leg.; host *Ledum palustre*; http://mus.utu.fi/ZMUT.TYPE798. ***Paratypes***: 10 females; Finland, Salla, Tuntsa; 67°18'11"N, 29°16'18"E; 01. Aug. 2019; Seppo & Christina Nokkala leg.; host *Ledum palustre*; http://mus.utu.fi/ZMUT.TYPE799. 5 females; Russia, Baikal; 51°54'25"N, 105°04'14"E; July 2007; E. Labina leg.; host *Ledum palustre*; http://mus.utu.fi/ZMUT.TYPE800. 6 females; Russia, Vorkuta; 67°30'00"N, 64°02'00"E; 6 Aug. 2013; N. Khabasova leg.; host *Ledum palustre*; http://mus.utu.fi/ZMUT.TYPE801. The holotype and paratypes are deposited at the Zoological Museum, University of Turku, Finland.

##### Description.

Adult coloration resembles *C. ledi*, but is more brownish with dark markings. Wings are yellowish and transparent with yellowish veins. Males are unknown. Overall length of females is similar to that of *C. ledi* (2.53–3.04 mm, N = 5).

##### Diagnosis.

**Figure 8.**
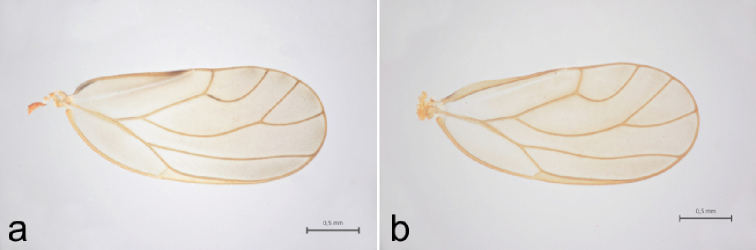
Comparison of forewing coloration in *C. borealis* sp. nov. and *C. ledi***a***C. borealis* sp. nov., dark brown apex and basal wing margin in clavus **b***C. ledi*, clavus without dark brown markings.

**Figure 9.**
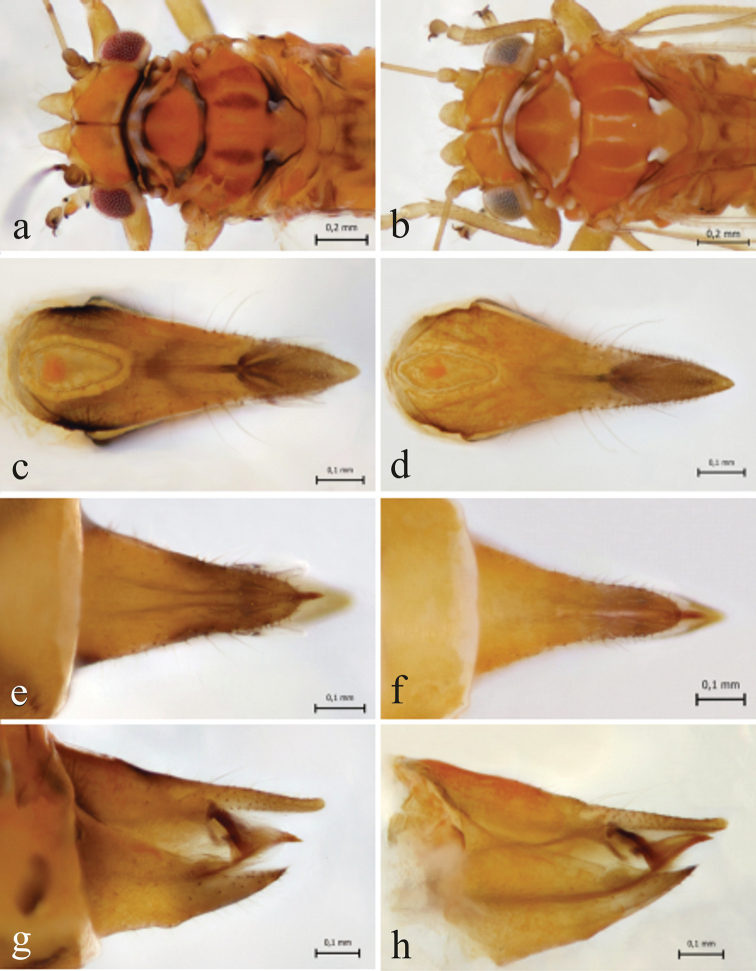
Comparison of morphological details between *C. borealis* sp. nov. (**a, c, e, g**) and *C. ledi* (**b, d, f, h**). Mesoscutum (**a–b**) dorsal plate (proctiger) (**c–d**) subgenital plate, ventral view (**e–f**) female terminalia in side view (**g–h**).

**Figure 10.**
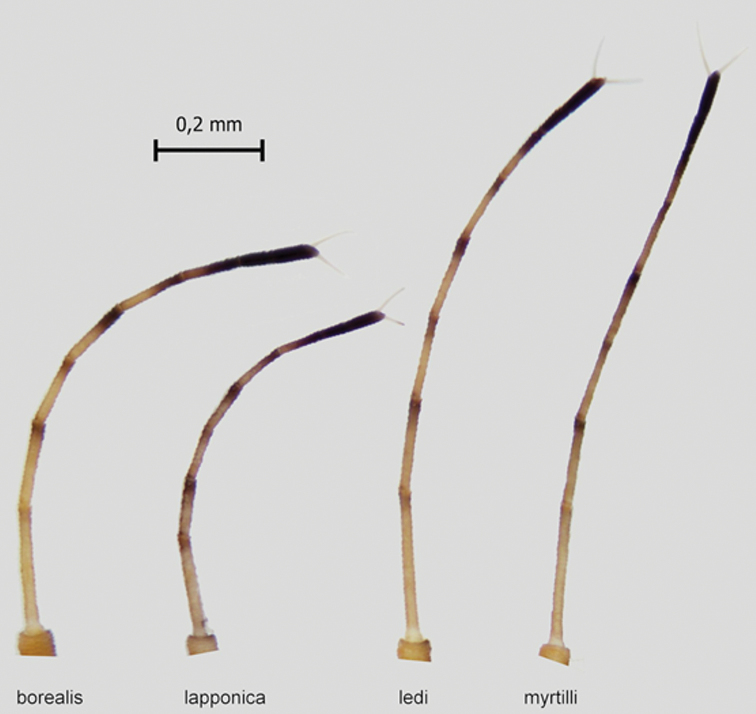
Antennae in *C. borealis* sp. nov., *C. lapponica* sp. nov., *C. ledi* and *C. myrtilli*.

The distribution of the surface spinules in the c+sc cell of forewing in *C. borealis* (Fig. 5b) is similar to spinule distribution in *C. ledi*. However, *C. borealis* and *C. ledi* can be indentified reliably by differences in their external morphology (Figs 8–10). A conspicuous difference is found in the forewings, where in clavus, apex and basal wing margin are dark brown in *C. borealis* (Fig. 8). Typically, on mesoscutum there are four highly pigmented longitudinal stripes in *C. borealis*, that are completely absent in *C. ledi* (Fig. 9a–b).

In female terminalia in dorsal view, the circumanal pore ring complex is wider in *C. borealis* than in *C. ledi* (Fig. 9c–d). The ventral subgenital plate seen from below narrows evenly towards the rounded apex in *C. borealis* while in *C. ledi* the structure is more slender and narrows strongly at first and then more evenly towards the apex (Fig. 9e–f). In *C. borealis*, the structure of the female terminalia in side view resembles that found in *C. myrtilli* and is quite different from that found in *C. ledi* (Fig. 9g–h). The antennae in *C. borealis* are thicker and shorter than in *C. ledi* (Fig. 10). Antennae are shortest in *C. lapponica* and thickness is similar compared to that of *C. borealis*, while the antennae have equal length in *C. myrtilli* and *C. ledi*.

##### Distribution.

*C. borealis* forms dense populations in northern Fennoscandia down to latitude 66° (Table 1.) It is also not uncommon to find mixed populations with *C. ledi*. It is easily understood as they are both parthenogenetic and live in those populations strictly reproductively isolated from each other. On the other hand, the wide distribution of *C. borealis* from Western Europe to Lake Baikal in the east suggests that *C. borealis* is of old origin. In some locations, most specimens in mixed populations living on *L. palustre* are *C. borealis*, as in Utsjoki, Ailigas (320 m), while in another location, in Utsjoki, Utsjoki Hietala near the sea level, the proportion of *C. ledi* is close to 20%. However, in several locations, as in Salla, Tuntsa and Salla, Niemelä, as well as in Kuusamo, Kantojoki and Kuusamo, Sakkojoki all individuals collected were *C. borealis*. The species *C. borealis* was found to be quite common in northern Finland. However, the hitherto known distribution of the species in Fennoscandia is restricted to north of latitude 63°.

##### Etymology.

The name “borealis” which means “north” or “northern” in Latin was given because of the wide Palearctic distribution of the species.

## Discussion

In the present study, we have described two new psyllid species, *C. lapponica* sp. nov. and *C. borealis* sp. nov. based on *COI* barcoding DNA sequence and uncorrected p-distance differences as well as morphological and cytological characteristics (Tables 2–3). The uncorrected p-distance differences between *C. lapponica* sp. nov. and the three species *C. borealis* sp. nov., *C. myrtilli* and *C. ledi* is of the same magnitude (> 3%) as the difference between the recognized species *C. myrtilli* and *C. ledi*. The difference between *C. lapponica* sp. nov. and *C. fraudatrix* is somewhat smaller or 2.04%. *C. lapponica* sp. nov. is a rare, bisexual alpine species with a high-altitude distribution in northern Fennoscandia, utilizing the same food plant and forming sympatric populations with *C. myrtilli*. However, phylogenetically it is associated most closely with another bisexual species, *C. fraudatrix* described from Poland, Bieszczady Mountains (Kuznetsova et al. 2012). Being rare and adapted to an open habitat, *C. lapponica* sp. nov. is very sensitive to exceptional environmental constraints, as dryness or heavy rains. This was realized during 2018, when late summer suffered from extremely warm and dry weather in northern Fennoscandia. Consequently, lower vegetation was completely dried out above tree line in both Utsjoki, Ailigas and Kilpisjärvi. Apparently, for this reason not a single specimen of *C. lapponica* was found in either of these locations. As *C. lapponica* sp. nov. is well adapted to an open habitat and harsh environment, it is plausible that the species might be found in similar habitats in former refugial areas in Central Europe. It is exceptional that a bisexual species, *C. lapponica* sp. nov. has migrated towards the north during recolonization after the last glacial period, while all other related recolonizing species, *C. myrtilli*, *C. ledi* and *C. borealis* sp. nov., are apomictic parthenogens (Nokkala et al. 2008, 2013, 2015, 2017).

**Table 3.** Summary of host plants, type of reproduction and morphological and cytological diagnostic characters among the *Cacopsylla* species studied.

**Species**	**Host plant**	**Type of reproduction**	**Ploidy level in parthenogenetic females**	**Karyotype**	**Type of meiosis in males**	**Number of follicles per testis**	**Reference**
*C. myrtilli*	*Vaccinium myrtillus*, *V. uliginosum*	apomictic parthenogenesis (with rare diploid, nonfunctional males)	triploid	3n = 36+XXX	achiasmate	4 (in rare males)	Nokkala et al. 2013
*C. borealis*	*Ledum palustre*	apomictic parthenogenesis	penta-ploid	5n = 60+XXXXX	–	–	Present study
*C. ledi*	*L. palustre*	apomictic parthenogenesis (with rare diploid males and females)	triploid	3n = 36+XXX	–	4 (in rare males)	Nokkala et al. 2017
*C. lapponica*	*V. uliginosum*	bisexual	–	2n = 24+X (males); 24+XX (females)	chiasmate	2	Present study
*C. fraudatrix*	*V. myrtillus*	bisexual	–	2n = 24+X (males); 24+XX (females)	chiasmate	2	Kuznetsova et al. 2012

On the other hand, *C. borealis* sp. nov. is an abundant pentaploid species with apomictic parthenogenetic reproduction, it has a wide palearctic distribution, may occur alone or form mixed populations with *C. ledi* on *L. palustre*. It is phylogenetically tightly associated with another parthenogen, *C. myrtilli*. In *COI* phylogeny, the parthenogenetic species *C. ledi* and *C. borealis* sp. nov. + *C. myrtilli* form a monophyletic sister clade to the clade consisting of bisexual *C. lapponica* sp. nov. + *C. fraudatrix*. All parthenognetic species form abundant populations north of latitude 63° in Fennoscandia. *C. borealis* sp. nov. has not been recorded south of this, populations of *C. myrtilli* are sparse, while *C. ledi* is relatively abundant, at least in southern parts of Finland around latitude 60° (Nokkala et al. 2017) and is found as south as Germany (Ossiannilsson 1992). It is remarkable, that the bisexual species *C. lapponica* sp. nov. has an extremely narrow distribution and habitat range compared to the three parthenogenetic species subjected in this study. In fact, these observations on the distribution ranges are well in accordance with the hypothesis of geographic parthenogenesis stating that parthenogenetic species have a wider ecological adaptation ability than their sexual progenitors (Kearney 2005).

In the present study, the two novel species, *C. lapponica* sp. nov. and *C. borealis* sp. nov., were found by chance while sampling the known species of *C. myrtilli* and *C. ledi*. It is plausible that there still exist undescribed species in northern high-altitude habitats waiting to be discovered. As climate change proceeds, northern habitats will experience substantial changes. This may lead to a situation, where an undescribed rare species with restricted habitat requirements will become extinct before it is found. Considering the species in the present study, *C. lapponica* sp. nov. is an example of a species endangered to become extinct, because of its narrow habitat requirements. Therefore, it is evident, that there is an urgent need to look systematically for undescribed species in northern high-altitude habitats.

## Conclusions

In the present study we have described two new psyllid species in the genus *Cacopsylla* Ossiannilssion, 1970, *C lapponica* sp. nov. and *C. borealis* sp. nov., based on chromosomal analyses, *COI* haplotype analysis and morphological characters. *C. lapponica* sp. nov. is bisexual and *C. borealis* sp. nov. a pentaploid parthenogenetic species. They may form sympatric populations with *C. myrtilli* and *C. ledi*, respectively. In *COI* phylogeny, *C. lapponica* sp. nov. is associated with another bisexual species, *C. fraudatrix*, while *C. borealis* sp. nov. associates with parthenogenetic *C. myrtilli* and *C. ledi*.

The authors have declared that no competing interests exist.

## Supplementary Material

XML Treatment for Cacopsylla lapponica

XML Treatment for Cacopsylla borealis
